# A Text-Book of Obstetrics

**Published:** 1902-01

**Authors:** 


A TEXT-BOOK OF OBSTETRICS. By Barton Cooke Hirst, M. D., Professor of Obstetrics in the University of Pennsylvania. Third edition, thoroughly revised and enlarged. Royal octavo, 873 pages, with 704 illustrations, many of them in colors. Philadelphia and London: W. B. Saunders & Co. 1901. Cloth, $5.00 net.

In his preface the author in a thoroughly characteristic manner pays respectful attention to his own professional record and attainments, which simply goes to show that modesty is not always associated with merit for, as teacher, writer and practical obstetrician, Dr. Hirst possesses all the merit be claims for himself.

It has never been our pleasure to examine a better written, better arranged or more splendidly illustrated work on obstetrics. In every way it bears evidence that it is the outcome of a large practical experience. It is clear, comprehensive and original, a credit alike to the author and to the University of Pennsylvania which, as Professor of Obstetrics, he ably represents. R.

